# Peripheral Anomalies in USH2A Cause Central Auditory Anomalies in a Mouse Model of Usher Syndrome and CAPD

**DOI:** 10.3390/genes12020151

**Published:** 2021-01-24

**Authors:** Peter A. Perrino, Dianne F. Newbury, R. Holly Fitch

**Affiliations:** 1 Department of Psychological Science/Behavioral Neuroscience, University of Connecticut, Storrs, CT 06269, USA; roslyn.h.fitch@uconn.edu; 2 Faculty of Health and Life Sciences, Oxford Brookes University, Oxford OX3 0BP, UK; diannenewbury@brookes.ac.uk

**Keywords:** central auditory processing disorder, Usher syndrome type 2, USH2A, superior olivary complex, medial geniculate nucleus

## Abstract

Central auditory processing disorder (CAPD) is associated with difficulties hearing and processing acoustic information, as well as subsequent impacts on the development of higher-order cognitive processes (i.e., attention and language). Yet CAPD also lacks clear and consistent diagnostic criteria, with widespread clinical disagreement on this matter. As such, identification of biological markers for CAPD would be useful. A recent genome association study identified a potential CAPD risk gene, *USH2A*. In a homozygous state, this gene is associated with Usher syndrome type 2 (USH2), a recessive disorder resulting in bilateral, high-frequency hearing loss due to atypical cochlear hair cell development. However, children with heterozygous *USH2A* mutations have also been found to show unexpected low-frequency hearing loss and reduced early vocabulary, contradicting assumptions that the heterozygous (carrier) state is “phenotype free”. Parallel evidence has confirmed that heterozygous *Ush2a* mutations in a transgenic mouse model also cause low-frequency hearing loss (Perrino et al., 2020). Importantly, these auditory processing anomalies were still evident after covariance for hearing loss, suggesting a CAPD profile. Since usherin anomalies occur in the peripheral cochlea and *not* central auditory structures, these findings point to upstream developmental feedback effects of peripheral sensory loss on high-level processing characteristic of CAPD. In this study, we aimed to expand upon the mouse behavioral battery used in Perrino et al. (2020) by evaluating central auditory brain structures, including the superior olivary complex (SOC) and medial geniculate nucleus (MGN), in heterozygous and homozygous *Ush2a* mice. We found that heterozygous *Ush2a* mice had significantly larger SOC volumes while homozygous *Ush2a* had significantly smaller SOC volumes. Heterozygous mutations did not affect the MGN; however, homozygous *Ush2a* mutations resulted in a significant shift towards more smaller neurons. These findings suggest that alterations in cochlear development due to *USH2A* variation can secondarily impact the development of brain regions important for auditory processing ability.

## 1. Introduction

Individuals diagnosed with central auditory processing disorder (CAPD) experience difficulties with multiple mechanisms that subserve acoustic information processing. These include, but are not limited to, sound localization, temporal discrimination, discrimination between two or more competing auditory stimuli, auditory pattern recognition and dichotic listening [1,2]. Moreover, affected individuals have difficulties with speech processing that include attending to verbal input (i.e., oral instruction) and comprehending complex sentences [3]. As a result, affected children often experience poor academic performance and reduced quality of life [4,5].

Nonetheless, there is ongoing debate within the audiology community as to the definition of—and diagnostic criteria for—CAPD. This includes whether CAPD should be considered a DSM-defined disorder. According to the American Speech and Hearing Association (ASHA), individuals clinically diagnosed with any of the aforementioned auditory impairments have clinically defined CAPD [1]. However, multiple other audiology groups (i.e., the American Academy of Audiology (2010) [6], the British Society of Audiology (2011) [4] and the Canadian Interorganizational Steering Group for Speech-Language Pathology and Audiology (2012) [7]) adopt different standards. The discrepancies across organizations include differences in phenotypic description, ascribed causal mechanisms and classification of co-morbidities (see [2] for a review). These disparities contribute to controversy in recognizing CAPD, with varied results in the attribution of symptoms to other disorders. For instance, Dawes and Bishop (2010) [8] reported that 52% of children diagnosed with CAPD could also fit a diagnosis of dyslexia, specific language impairment, or both. Children with CAPD have also been shown to meet behavioral profiles for attention deficit disorder [9,10], suggesting CAPD may resemble a more general cognitive disorder rather than an auditory perception disorder.

The lack of a clear causal genetic, peripheral or neurologic mechanism adds another layer of difficulty to defining CAPD. Ongoing research is crucial to determining whether CAPD is the result of poor auditory processing and/or integration with higher-order cognitive processes, subclinical hearing impairments that affect cochlear development, comorbid cognitive disorders (as discussed above), or a combination of factors. Additionally, genetic contributions to CAPD remain understudied [11]. Brewer et al. (2016) [12] reported that auditory processing skills (i.e., temporal processing and pitch discrimination) subserving the perception of spoken language are heritable. As such, it is possible that auditory processing difficulties seen in individuals with CAPD arise from genetic variants and/or mutations.

One promising CAPD-risk gene is *USH2A*, which is clinically associated with Usher syndrome type 2 (USH2; [13]). Individuals with USH2 experience bilateral hearing loss at high frequencies and retinitis pigmentosa beginning at puberty [14,15]. USH2 results from homozygous loss-of-function of *USH2A*, with heterozygous individuals considered to be unaffected carriers [16,17]. The *USH2A* protein is expressed primarily in the cochlea and retina but not in the brain, meaning that USH2 is considered a peripheral disorder [18]. Usherin plays a critical role in cochlear hair cell maturation and acts to connect developing stereocilia with kinocilium via a transient lateral ankle link that helps guide developing hair cells into their proper orientation [19,20]. Lui et al. (2007) [19] reported that the outer hair cells of the basal cochlea were missing in mice with a homozygous knockout of *Ush2a* (the rodent homolog of *USH2A*), consistent with high-frequency hearing loss in individuals with USH2.

While it is well established that homozygous mutations of *USH2A* cause UHS2, little is known about how heterozygous mutations affect hearing ability or auditory processing. Historically, heterozygous mutations of *USH2A* have been considered nonpathogenic, with such individuals classified as “unaffected carriers” of USH2. Yet several studies report low-frequency hearing loss or sensorineural abnormalities in USH2 carriers [20,21,22,23]. As a result of abnormalities reported in USH2 carriers, researchers have recently become interested in how heterozygous mutations of *USH2A* might contribute to auditory processing ability, including disorders like CAPD.

To study the relationship between heterozygous *USH2A* mutations, CAPD and language outcomes, Perrino et al. (2020) [24] sought to combine human whole genome sequencing with mouse model behavioral phenotyping. Specifically, we conducted genome sequencing of a family with individuals affected by a severe expressive language disorder, as well as phenotypic characteristics of CAPD (i.e., difficulties understanding oral instructions). Affected family members were found to have a heterozygous stop-gain mutation in the *USH2A* gene (NP_996816:p.Gln4541*), suggesting that the heterozygous *USH2A* mutation might have caused the auditory processing deficits in affected family members. To further test this hypothesis, we evaluated *Ush2a* heterozygous (HT) mice on a battery of rapid auditory processing tasks. We found that HT mice had low-frequency hearing impairments, which subsequently contributed to higher-order auditory processing difficulties that persisted even when hearing deficits were covaried out. Importantly, simultaneous testing showed that *Ush2a* KO mice were affected by significant high-frequency auditory processing impairments, a defining characteristic of USH2. These low-level hearing deficits in homozygous mice also contributed to higher-order auditory processing difficulties reflective of central mechanisms. Human genome-wide association studies (GWAS) also suggest that heterozygous *USH2A* mutations contribute to a CAPD phenotype. Specifically, though the same stop-gain mutation that was reported in our discovery family was not present in the UK10K dataset [25], children with pathogenic heterozygous *USH2A* variants nonetheless showed low-frequency hearing impairments (i.e., increased low-frequency hearing thresholds (+1.2 dB HL at 500 Hz)), as well as reductions in vocabulary, when compared to children without an *USH2A* mutation [24]. These results were highly novel in identifying heterozygous *USH2A* mutations as a CAPD risk, with impacts on low-frequency hearing as well as higher-order auditory processing abilities necessary for typical language and communication development.

This current study builds upon Perrino et al. (2020) [24]. Here, using *postmortem* brains from the behaviorally evaluated mice, we analyzed the neuroanatomical consequences of *Ush2a* genetic variations. We hypothesized that, despite the lack of *Ush2a* expression in the CNS, anatomical anomalies may be evident in the overall volume, neuron size and/or neuronal population in brain structures that subserve central auditory processing (i.e., the medial geniculate nucleus (MGN) and superior olivary complex (SOC)) in both heterozygous and knockout subjects. We predicted differing anatomical anomalies between heterozygous (HT) and homozygous (KO) subjects, given that HT mutations affect low-frequency processing and KO mutations affect high-frequency processing. Results from volumetric analysis showed a significant increase in right SOC volume for *Ush2a* HT mice, coupled with a significant decrease in right SOC volume for *Ush2a* KO mice. Within the right MGN, we found a significant shift towards more smaller neurons in *Ush2a* KO mice, while HT mice were unaffected. Together, our results suggest that altered cochlear development impacts higher-order auditory processing at both a functional and structural level, but differently so in *Ush2a* HT and KO subjects. These anomalies could account for complex auditory and speech processing impairments observed in some individuals with CAPD, as well as those with Usher syndrome type 2.

## 2. Materials and Methods

### 2.1. Subject Generation

Six homozygous *Ush2a* male subjects (F1 generation) were rederived on an 129S4/SvJaeJ background strain at the Center for Mouse Genome Modification (previously known as the Gene Targeting and Transgenic Facility) at UConn Health via genetic material obtained from Dr. Jun Yang (University of Utah; [19]). These six male *Ush2a* KO mice were crossed with six wildtype (WT) control mice (29S4/SvJaeJ; stock number 009104) obtained from the Jackson Laboratory (Bar Harbor, ME) to generate an all heterozygous (HT) F2 generation. To generate experimental (F3) subjects, HT × HT breeding pairs from the F2 generation were established, resulting in litters containing all three genotypes (homozygous, heterozygous and wildtype). Following weaning (postnatal day (P) 21), ear punches were collected from each subject and used for genotyping via PCR (DNA primer information can be found in [19]). After puberty (P40), subjects from the F3 generation were randomly selected and used for behavioral testing and histological assessment. Additionally, at this time, experimental subjects were single housed in standard Plexiglass mouse chambers (12 h/12 h light–dark cycle) with food and water available *ad libitum*. The subjects used here, as well as the breeding information, are the same as used in Perrino et al. (2020) [24].

### 2.2. Behavioral Testing

Beginning at P65, subjects were assessed on a battery of auditory processing tasks aimed to evaluate the subject’s ability to process and discriminate complex acoustic information relevant to communication ability (see [26] for review). For a complete description of the behavioral battery each subject underwent, see [24]. In short, acoustic processing ability was assessed using a modified prepulse inhibition (PPI) paradigm in which the subject’s acoustic startle response was measured following the presentation of a loud (105 dB, 50 ms) startle eliciting stimulus (SES; 1000–10,000 Hz broadband burst) (“uncued” trials). During each testing session, acoustic cues were pseudorandomly presented before the SES—the subject’s acoustic startle response during “cued” trials was measured. If the subject was able to detect the acoustic cue—the goal of each auditory processing task—their acoustic startle response should have been reduced (or attenuated) as the cue informs the subject that the SES is about to occur. The difference in startle response during “uncued” and “cued” trials can be calculated as an “attenuation score”—a ratio of [average “cued” startle response/average “uncued” startle response] × 100. The lower the attenuation score, the better the subject’s performance in detecting the cue. Subjects with an attenuation score of 100% were deemed to have not detected the cue, as their “uncued” and “cued” startle responses were similar.

Each subject underwent a variety of acoustic processing tasks, each designed to assess a different aspect of acoustic processing ability. Subjects were first evaluated on a normal single tone (NST) task that used a simple pure tone cue to assess baseline hearing ability, typical acoustic startle response (i.e., motor ability), and prepulse inhibition. Attenuation scores for the NST task were used as a covariate for subsequent auditory processing tasks to eliminate individual differences. More complex auditory processing tasks were used to evaluate spectral, temporal, or both spectral and temporal (spectro-temporal) aspects of auditory processing ability. For example, embedded tone (EBT) consisted of a pure tone background and an auditory cue that was different than the pure tone background and varied in duration. Pitch discrimination (PD) consisted of a pure tone background and an acoustic cue that had a fixed duration but varied in frequency. Additionally, each task was presented in both a sub-ultrasonic and an ultrasonic frequency range. The use of multiple auditory processing tasks, combined with the use of multiple frequency ranges, allows for the detailed evaluation of how *USH2A* mutations affect different aspects of acoustic processing ability. See [24] for a description of each task used.

### 2.3. Histology

Following the completion of behavioral testing (P150) and after being weighed, subjects were anesthetized using ketamine (100 mg/kg) and xylazine (15 mg/kg) and transcardially perfused using a 0.9% saline solution followed by 10% formalin. The brains were postfixed in 10% formalin following extraction. Each brain was serially and coronally sectioned (60 µm) using a Leica VT1000 S vibratome (Leica Biosystems Inc., Buffalo Grove, IL, USA). Olfactory bulbs were removed using a surgical blade and the flat surface that remained was glued to the vibratome stage—slicing began at the cerebellum and progressed towards the frontal cortex (posterior → anterior). Every coronal section of the brain was mounted to a gelatin-subbed glass slide until the cerebellum was completely sectioned. This methodology was performed to ensure the complete sectioning of the superior olivary complex. Sectioning continued past the cerebellum and every second section was mounted on a gelatin-subbed glass slide. All slides were subjected to cresyl violet to stain for Nissl bodies. Slides were then cover-slipped with DPX mounting medium.

### 2.4. Stereological Measurements

**Figure 1:**
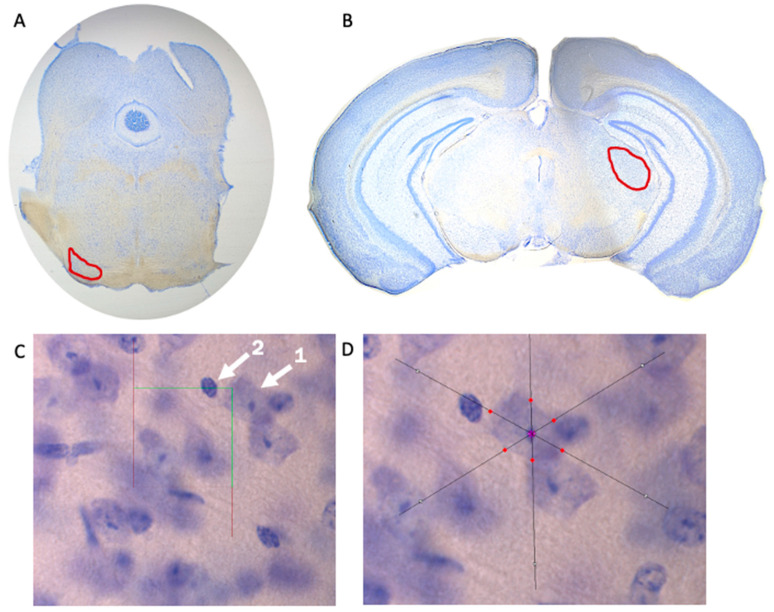
Stereological Measurements. Visual representation of the superior olivary complex (SOC) (**A**) and medial geniculate nucleus (MGN) (**B**) taken at 2.5× magnification to determine volume of brain region. (**C**) The Optical Fractionator probe of Stereo Investigator was used to determine neuron population. Arrow 1 indicates a neuron that was counted due to the presence of a clearly defined nucleolus, while Arrow 2 indicates a cell that was not counted. (**D**) The Nucleator probe was used to determine neuron size (area). Optical Fractionator and Nucleator probes were used at 100× magnification.

Brain tissue underwent stereological analysis via Stereo Investigator (MBF Biosciences, Williston, VT, USA) using a Zeiss Axio Imager A2 microscope (Carl Zeiss, Thornwood, NY, USA). Superior olivary complex (Figure 1A) and medial geniculate nucleus (Figure 1B) volumes were estimated using the Cavalieri Estimator probe, neuron population was estimated using the Optical Fractionator probe (Figure 1C), and the Nucleator probe was used to measure neuronal cell area (Figure 1D). Measurements within the SOC were performed at a sampling frequency of every section (across eight total sections), while measurements within the MGN were performed with a sampling frequency of every second section (across six total sections). Contours to define each region and to provide volumetric estimates were determined via stereotaxic atlas [27] and drawn at 2.5× magnification. All other stereological measurements (i.e., neuron population and neural cell area) were evaluated at 100× magnification. A sampling grid of 150 µm × 150 µm and a 30 µm × 30 µm counting frame was selected for the SOC, while a sampling grid of 225 µm × 225 µm and a 25 µm × 25 µm counting frame was selected for the MGN. Neurons were defined as having one distinct nucleolus within the nucleus—glial cells or other cell types within the brain were not counted (Figure 1C).

### 2.5. Statistical Analysis

Genotype differences for the volume of each region (i.e., SOC and MGN), neuron population within each region, and average neuronal cell area within each region, were analyzed using univariate ANOVAs. Additionally, univariate ANOVAs were performed between each Genotype to determine how heterozygous *Ush2a* mutations differed from homozygous mutations—a necessary analysis for determining how each mutation contributes to the behavioral differences between HT and KO subjects (low-frequency vs. high-frequency auditory processing). To evaluate how *Ush2a* mutations affect neuronal cell size (area) distribution, the Kolmogorov–Smirnov (K–S) test was conducted on the cumulative percent distribution for each Genotype. Analyses were conducted for the left and right hemispheres, as well as both together (i.e., total SOC or total MGN). All univariate ANOVAs and correlational analyses were conducted via the car package [28] in R (v3.4.4; [29]). A total of 21 subjects were used in the histological assessment of the SOC (WT, n = 7 (one subject dropped due to poor tissue integrity); HT, n = 7; KO, n = 7) and 22 subjects for the histological assessment of the MGN (WT, n = 8; HT, n = 7; KO, n = 7).

### 2.6. Ethics

All animal procedures were approved by the University of Connecticut’s Institute for Animal Care and Use Committee (IACUC; Protocol No. A18-050) and followed the Guide for the Care and Use of Laboratory Animals [30]. This study was designed to comply with ARRIVE guidelines [31].

## 3. Results

### 3.1. SOC Volumetric Analysis

**Figure 2:**
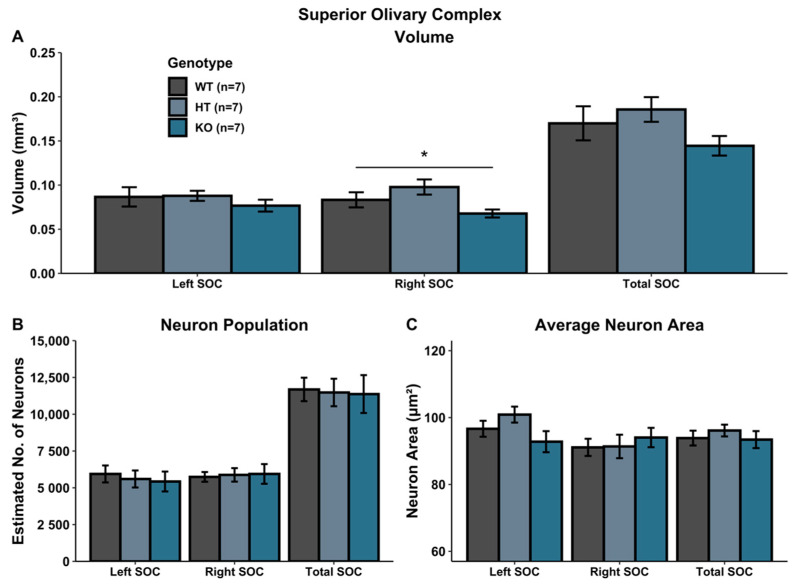
Histological assessment in SOC. (**A**) Volumetric analysis of left, right and total (left + right) SOC. Heterozygous *Ush2a* mutations increased the volume of the right SOC while homozygous *Ush2a* mutations reduced the volume of the right SOC. (**B**,**C**) There were no significant genotype differences in neuron population (**B**) or average neuron area (**C**). * *p* < 0.05.

A univariate ANOVA comparing SOC volume revealed a main effect of Genotype in the right SOC [right: *F*(2, 18) = 4.034, *p* < 0.05], reflecting a volumetric increase in HT subjects relative to WTs, coupled with a volumetric decrease in KO subjects (HT vs. KO; *F*(1, 12) = 9.558, *p* < 0.05).There was no significant Genotype effect in the left SOC [*F*(2, 18) = 0.568, *p* > 0.05], nor for the total SOC [*F*(2, 18) = 1.874, *p* > 0.05] (Figure 2A).

### 3.2. MGN Volumetric Analysis

**Figure 3:**
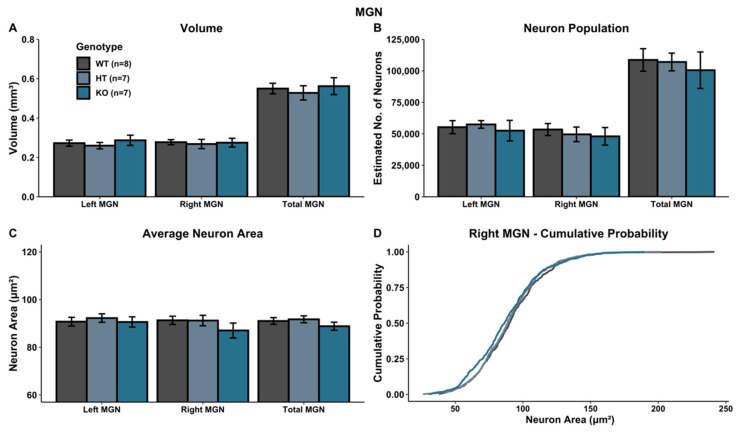
Histological assessment in MGN. (**A**–**C**) There were no significant genotype differences when evaluating volume (**A**), neuron population (**B**) or neuron area (**C**). (**D**) Comparison of cumulative percent distribution of neuronal cell size (area) revealed a significant shift towards fewer larger neurons and more smaller neurons in the right MGN in *Ush2a* homozygous (KO) mice.

A univariate ANOVA comparing MGN volume revealed no main effect of genotype in either hemisphere (left: *F*(2, 19) = 0.468, *p* > 0.05; right: *F*(2, 19) = 0.0598, *p* > 0.05; total: *F*(2, 19) = 0.232, *p* > 0.05) (Figure 3A).

### 3.3. SOC Cellular Analysis

There was no significant Genotype effect when evaluating neuron population within the SOC (left SOC: *F*(2, 18) = 0.183, *p* > 0.05; right SOC: *F*(2, 18) = 0.040, *p* > 0.05; total SOC: *F*(2, 18) = 0.028, *p* > 0.05) (Figure 2B). Additionally, there was no Genotype effect on average neuron size (area) in the SOC (left SOC: *F*(2, 18) = 2.3048, *p* > 0.05; right SOC: *F*(2, 18) = 0.290, *p* > 0.05; total SOC: *F*(2, 18) = 0.437, *p* > 0.05) (Figure 2C).

### 3.4. MGN Cellular Analysis

There was no significant Genotype effect on neuron population within the MGN (left MGN: *F*(2, 19) = 0.174, *p* > 0.05; right MGN: *F*(2, 19) = 0.174, *p* > 0.05; total MGN: *F*(2, 19) = 0.168, *p* > 0.05) (Figure 3B), nor for average neuron size (area) within the MGN (left MGN: *F*(2, 19) = 0.219, *p* > 0.05; right MGN: *F*(2, 19) = 1.039, *p* > 0.05; total MGN: *F*(2, 19) = 0.965, *p* > 0.05) (Figure 3C). Additionally, in the left MGN, no significant K–S statistics for the cumulative distribution of cell size were seen (left MGN (WT vs. HT): *p* > 0.05; left MGN (WT vs. KO): *p* > 0.05; left MGN (HT vs. KO): *p* > 0.05). However, within the right MGN, WT and KO subjects were significantly different (right MGN (WT vs. KO): *p* < 0.05), with a shift towards more smaller neurons in KO subjects. WT vs. HT subjects did not yield a significant K–S statistic (right MGN (WT vs. HT): *p* > 0.05), nor did HT vs. KO subjects (right MGN (HT vs. KO): *p* > 0.05) (Figure 3D). No effects were seen for the overall MGN (total MGN (WT vs. HT): *p* > 0.05; total MGN (WT vs. KO): *p* > 0.05; total MGN (HT vs. KO): *p* > 0.05).

## 4. Discussion

The current study was designed to neuroanatomically evaluate the central auditory consequences of heterozygous and homozygous mutations in an *Ush2a* mouse model. The study was based on human clinical evidence that homozygous mutations of *USH2A* result in Usher syndrome type 2 [13], as well as recent evidence that heterozygous *USH2A* mutations may be a genetic risk factor for CAPD [24]. Since anomalies in auditory processing represent core features of both CAPD and USH2 (though with very different functional profiles; [3,14]), central auditory structures of the superior olivary complex and medial geniculate nucleus were evaluated. Results showed that heterozygous *Ush2a* mutations resulted in an increase in right SOC volume, while homozygous *Ush2a* mutations resulted in a decrease in right SOC volume, as well as a shift towards fewer large and more small neurons in the right MGN. To the best of our knowledge, these results are the first to report neuroanatomical anomalies in a mouse model of either CAPD or Usher syndrome type 2. The results are particularly exciting given a lack of usherin expression in the brain [18], which suggests substantial developmental effects of peripheral auditory anomalies on the central auditory system.

### Neuroanatomical Differences between HT and KO Subjects

The two structures evaluated in this study, SOC and MGN, both play an important role in the central auditory system (see [32] for review). The SOC is one of the first stops for ascending auditory information, primarily mediating sound localization via the convergence of binaural sensory input [33,34]. To our knowledge, there are not significant processing differences between right and left SOC, both of which receive input from ipsilateral and contralateral cochlea [35]. Here, we report that the right SOC is smaller in KO subjects, and larger in HT subjects. In considering possible mechanisms for these anomalies, Liu et al. (2007) [19] reported that mice with a homozygous *Ush2a* deletion had an absence of outer hair cells in the basal cochlea, an area primarily responsible for the detection of high-frequency auditory information. This cochlear abnormality could have contributed to the observed reductions in right SOC volume, since regions that respond to high-frequency auditory information are presumably receiving anomalous/degraded sensory input. The notion that anomalous brain development may result from altered or absent sensory input is well established and has been studied in multiple sensory modalities, including the auditory system [36,37,38,39,40,41]. These SOC reductions in KO subjects may have further contributed to the high-frequency processing impairments reported by Perrino et al. (2020) [24]. The increase in SOC volume in subjects with heterozygous *Ush2a* mutations was surprising. However, there is ample evidence that atypical structural increases in the CNS can cause functional impairments (e.g., macrocephaly). Future studies are needed to (1) evaluate cochlear development and organization in heterozygous *Ush2a* mutant mice, and (2) determine how non-neuronal cell types (i.e., glial cells) might contribute to the volumetric differences reported here. It is possible that changes in glial morphology within the SOC in HT and/or KO mice contributed to the observed behavioral phenotypes. Nonetheless, the increase in right SOC volume in HT subjects provides evidence that altered sensory input can impact CNS development, as well as evidence that underlying neurologic anomalies may exist in CAPD.

In addition to the SOC, we assessed the MGN, a thalamic nucleus responsible for auditory processing. The MGN has been shown to be affected in other language- and communication-neurodevelopmental disorders; for example, Galaburda et al. (1994) [42] reported a shift towards more smaller MGN neurons in the brains of individuals with dyslexia. These initial findings of atypical MGN morphology led to further studies with animal models using induced mutations of dyslexia-risk genes and induced neuronal migration abnormalities. Both models showed anomalous MGN anatomy [43,44,45]. Importantly, atypical MGN development has been shown to impact auditory processing ability [46,47,48], which is a fundamental skill necessary for language development, as well as a good predictor of later language outcomes [49]. Within the autism spectrum disorders (ASD) population, for example, reductions in MGN volume [50] and altered thalamocortical connectivity [51] have been reported, and similar MGN anomalies have been observed in genetic mouse models of ASD [52], a disorder frequently characterized by anomalous language development and language impairments. Our findings that homozygous *Ush2a* mutations shift the cell size distribution towards fewer large and more small neurons in the right MGN further substantiate the potential role of MGN in language functions [53].

Finally, it is important to note that effects were observed explicitly in the right SOC and right MGN in both HT and KO mice. Given overwhelming evidence of left hemisphere lateralization for language and underlying auditory temporal processing [54,55,56], these results may seem puzzling. However, it is important to note that recent mouse research has shown evidence of left hemisphere lateralization in A1 specifically for processing of ultrasonic vocalizations and other spectro-temporal acoustic information, while right A1 may play a stronger role in frequency-based processing [57]. Although lateralization of MGN was not observed in this study, it is nonetheless possible that feedback effects of altered frequency-specific input could have selective effects on the projecting pathways to right A1, including the right SOC and MGN.

## 5. Conclusions

The goal of the current study was to provide a histological follow-up to Perrino et al. (2020) [24] by evaluating the consequences of heterozygous and homozygous *USH2A* mutations on central auditory structures in a transgenic mouse model. We report that *Ush2a* HT mice, a putative mouse model for CAPD, exhibited significantly increased right SOC volumes. Conversely, *Ush2a* KO mice—a well-accepted mouse model for USH2—exhibited significantly decreased right SOC volumes, and a shift towards smaller right MGN neurons. These neuroanatomical abnormalities may contribute to the low-frequency auditory processing impairments seen in HT mice, as well as associated language and communication impairments seen in individuals with pathogenic, heterozygous *USH2A* variants. Subcortical anomalies may also contribute to the high-frequency auditory processing impairments seen in KO mice, corresponding to clinical USH2 symptoms. Importantly, given evidence of usherin expression in the cochlea but not the brain, our results indicate: (1) an upstream impact of altered cochlear function on the central auditory system and (2) that the impacts differ for heterozygous and homozygous *Ush2a* mutations, commensurate with different hearing profiles. Future studies will be important in assessing additional central auditory structures in these mouse models (e.g., inferior colliculus, A1). Taken together, our findings strongly advocate for early genetic screening as a tool for detecting hearing and auditory processing disorders that may impact subsequent language development, and add to evidence from Perrino et al. (2020) [24] that *USH2A* carriers are not “phenotype-free”.

## Data Availability

The data presented in this study are available on request from the corresponding author.
